# The Weather and the Health of the City

**Published:** 1854-09

**Authors:** 


### THE WEATHER, AND THE HEALTH OF THE CITY.

As we wrote at the begining of August we have to write still—there is no abatement of the intense heat which has rendered this season so memorable. The mercury continues, at the end of the first week in September, to rise to blood heat every day. The nights are also very oppressive.

From the meteorological table prepared by L. YOUNG, Esq., which will be found on another page, the reader will gather what a month was August, 1854, in the neighborhood of Louisville. Mr. YOUNG's fine farm is nine miles east of the city. On the first and last days of the month the thermometer rose to 100 deg., and to 101, 102½ and 103 degrees on intermediate days. We are quite sure we are safe in saying, that no one ever before saw the temperature so high in Kentucky. There were three falls of rain during the month, two of which were very slight; one on the 27th of one inch; less than two inches for the month. For the preceding month the rains were 1.60 inches, or some two and a half inches for the two months during which our most important crops are maturing. Such a temperature and drouth are very extraordinary, but until the crops are harvested it is impossible to judge with precision how disastrous the season has been.

The rain on the 27th of August was preceded in Louisville by a violent hurricane, which did great damage to buildings and caused the loss of many lives. The morning was hot and sultry—98 deg. at 11 o'clock—and the clouds afforded indication of rain. About 11½ o'clock denser clouds began to gather over the city, and a little before 12 M., the storm broke upon it in all its fury. The direction of the wind was from south-west to north-east, and its chief violence was felt from 12th to 9th streets, between which most of the houses sustained more or less injury. The Presbyterian church on the corner of 11th and Walnut streets, in an unfinished condition, was prostrated, and about sixty persons buried beneath its ruins, one-third of whom were killed, and many others severely injured. The walls of the church fell inwards, and the roof following, the joists of the floor were broken through, and all were precipitated upon the congregation worshiping in the basement room below. Persons in the neighborhood describe the clouds which swept over the church as presenting the appearance of a water spout. The roof of the building was seen to rise two or three times before the walls gave way.

The relations of this remarkable condition of the seasons to etiology, are interesting. Health has prevailed to an extraordinary extent. It is the belief of some of the old practitioners of Louisville that so healthy a season was never before experienced in the city. All sources of malaria seem to have been dried up by the scorching sun. There is no moisture to favor vegetable decomposition any more than vegetable growth. The Ohio river is said to be lower than it has before been known to be in the last twenty years.

These facts are put upon record for the use of the future medical historian. They are certainly remarkable.

